# Supplementary material for: Is project-based learning effective among kindergarten and elementary students? A systematic review
Source: PLoS One. 2021 Apr 2;16(4):e0249627. doi: 10.1371/journal.pone.0249627 (PMC8018635; doi:10.1371/journal.pone.0249627)
Supplement: Attachment — Submitted filename: PONE-D-20-21356_R2_response.docx [file pone.0249627.s002.docx]

| **Comments made by Reviewer 1** | **Response / Changes to the manuscript** |
| --- | --- |
| 1. Please write the year of the selection paper in both the abstract and the manuscript. | Thanks for this suggestion. We have now mentioned the year of the literature search in the abstract and the main text. |
| 2. Please write the research sample background (outlining in which areas). | Thank you for the recommendation. The current version of Table 1 includes additional information about the knowledge area addressed and the background of the sample. |
| 3. Please describe the originality/value of this research.  4. Please detail the effectiveness of project-based learning for kindergarten and elementary school students regarding their academic performance in the introduction. | Note that this is exactly the question that we address in the present study: Whether there is sufficient evidence to support the claim that PjBL is effective at this stage. In the introduction we highlight that there is still no systematic review focused on the efficacy of PBL at the two basic levels of education addressed in the present study (kindergarten and elementary). The aim of the present review was to cover this gap. We hope that the changes made in the introduction (pp. 6-7) will make this clearer in the present version of the ms. Furthermore, our results also show that the evidence available so far is insufficient to draw any firm conclusion about the effectiveness of PjBL in this sample. We now state this more clearly in the Discussion. |
| **Comments made by Reviewer 2** | **Response / Changes to the manuscript** |
| 1. The authors could consider using “PjBL” to refer to project-based learning throughout the manuscript, because “PBL” often refers to problem-based learning in the literature. | Thank you for your suggestion. We have modified this acronym throughout the ms. |
| 2. I was wondering whether PjBL is suitable for kindergarten students. | As explained above, the main conclusion of our study is that without more and better evidence it is difficult to draw any firm conclusion about the effectiveness of PjBL in this population. We now say this explicitly in the Discussion. |
| 3. For the topics of effects of PjBL, I would suggest the authors to review a meta-analysis article: <https://doi.org/10.1016/j.edurev.2018.11.001> , for greater understanding of its effects by cutting-edge research.  4. This research only includes 9 articles; however, it seems that the above meta-analysis included 8 elementary school PjBL studies (many are different from yours). I suggest checking those studies (to include more articles). | Thank you for your suggestion. In the present version of the ms we discuss this meta-analysis and we have also used it as an additional source of primary studies. |
| 5. I suggest searching more databases such as EBSCOhost and ProQuest. | Searches with EBSCOhost have not returned any additional study. In previous meta-analyses of other educational interventions we have explored the grey literature with ProQuest and GoogleScholar to ameliorate the impact of publication bias on the results. We see the relevance of doing this in a meta-analytic review. But in the absence of a meta-analysis, we do not think that this literature will add substantial information. Note that our goal is to assess the methods and reporting quality of the best evidence available so far. We doubt we can find studies with higher methodological and reporting standards in the grey literature than among peer-reviewed academic journals. |
| 6. There is a gap between the 34,246 studies and the 38 studies (p. 7). | In the present version of the ms we explain explicitly that the screening of titles and abstracts allowed us to conclude that 34,208 studies did not meet the inclusion criteria. |
| 7. High resolution figures are needed; kindly provide high-resolution original figures (Figures 1–3) in the manuscript. | The online editorial manager automatically reduces the quality of the figures attached to the ms. But all the original figures have a resolution of 300 dpi, following the requirements established by PLoS ONE (300-600 dpi). |
